# Exosome-mediated transfer of miR-222 is sufficient to increase tumor malignancy in melanoma

**DOI:** 10.1186/s12967-016-0811-2

**Published:** 2016-02-24

**Authors:** Federica Felicetti, Alessandra De Feo, Carolina Coscia, Rossella Puglisi, Francesca Pedini, Luca Pasquini, Maria Bellenghi, Maria Cristina Errico, Elena Pagani, Alessandra Carè

**Affiliations:** Department of Hematology, Oncology and Molecular Medicine, Istituto Superiore di Sanità, Viale Regina Elena 299, 00161 Rome, Italy; Laboratory of Molecular Oncology, Istituto Dermopatico DELL’IMMACOLATA-IRCCS, 00167 Rome, Italy

**Keywords:** (da 3 a 10): Melanoma, MicroRNA, Exosomes, miR-222, PI3K/AKT

## Abstract

**Background:**

Growing evidence is showing that metastatic cell populations are able to transfer their characteristics to less malignant cells. Exosomes (EXOs) are membrane vesicles of endocytic origin able to convey their cargo of mRNAs, microRNAs (miRs), proteins and lipids from donors to proximal as well as distant acceptor cells. Our previous results indicated that miR-221&222 are key factors for melanoma development and dissemination. The aim of this study was to verify whether the tumorigenic properties associated with miR-222 overexpression can be also propagated by miR-222-containing EXOs.

**Methods:**

EXOs were isolated by UltraCentrifugation or Exoquick-TC^®^ methods. Preparations of melanoma-derived vesicles were characterized by using the Nanosight™ technology and the expression of exosome markers analyzed by western blot. The expression levels of endogenous and exosomal miRNAs were examined by real time PCR. Confocal microscopy was used to evaluate transfer and uptake of microvesicles from donor to recipient cells. The functional significance of exosomal miR-222 was estimated by analyzing the vessel-like process formation, as well as cell cycle rates, invasive and chemotactic capabilities.

**Results:**

Besides microvesicle marker characterization, we evidenced that miR-222 exosomal expression mostly reflected its abundance in the cells of origin, correctly paralleled by repression of its target genes, such as p27Kip1, and induction of the PI3K/AKT pathway, thus confirming its functional implication in cancer. The possible differential significance of PI3K/AKT blockade was assessed by using the BKM120 inhibitor in miR-222-transduced cell lines. In addition, in vitro cultures showed that vesicles released by miR-222-overexpressing cells were able to transfer miR-222-dependent malignancy when taken-up by recipient primary melanomas. Results were confirmed by antagomiR-221&222 treatments and by functional observations after internalization of EXOs devoid of these miRs.

**Conclusion:**

All together these data, besides generally confirming the role of miR-222 in melanoma tumorigenesis, supported its responsibility in the exosome-associated melanoma properties, thus further indicating this miR as potential diagnostic and prognostic biomarker and its abrogation as a future therapeutic option.

**Electronic supplementary material:**

The online version of this article (doi:10.1186/s12967-016-0811-2) contains supplementary material, which is available to authorized users.

## Background

Malignant melanoma is the most aggressive form of skin cancer whose incidence is doubling almost every 10 years. Although surgical resection is mostly a definitive treatment at the primary stages of the disease, the survival of patients is strongly reduced after metastatic dissemination, mostly due to resistance to conventional therapies [1, 2]. Therefore it is important to use new molecular approaches in order to better understand the mechanisms underlying melanoma progression.

MicroRNAs (miRs) are non-coding RNAs that regulate gene expression mostly at post-transcriptional level playing important roles in nearly all the biological functions, including tumor development and dissemination [3]. These small non coding RNAs have revealed a great potential as early diagnosis markers being highly stable and able to discriminate different subtypes of cancer [4]. Moreover the profile of circulating miRs was shown to reflect the expression pattern of tumor tissues [5].

Our previous results showed that miR-221&222 are key factors for melanoma development and dissemination, as they control the progression of the neoplasia through the down-modulation of several direct targets, as p27Kip1/CDKN1B, c-KIT receptor and c-FOS, all playing antineoplastic functions. Accordingly the miR-221&222-dependent repression leads to enhanced proliferation as well as differentiation and apoptosis blockade in melanoma cells [6, 7].

Growing evidence is showing that miRs are not strictly cellular, but are secreted in extracellular compartments, as body fluids as well as cell culture media, where they might be transported by vesicle and non vesicle carriers, including “exosomes” (EXO) [8]. EXOs are nanovesicles of diameter ranging between 50 to 140 nm, distinguished from other cell-derived vesicles by their origin, size, morphology and composition [9]. In fact, due to their endosomal origin, EXOs are enriched of certain proteins, including members of the tetraspanin family (CD9, CD81, CD82, CD63), heat shock proteins (Hsp60, Hsp70, Hsp90) and proteins of the multivesicular bodies (annexins, Rab GTPases and endosomal sorting complexes required for transport (ESCRT) proteins) [10]. EXOs secreted by tumor cells were shown to transfer oncogenic properties via horizontal propagation of mRNAs, miRs and proteins. More important, upon their release in the extracellular environment, EXOs are utilized by tumors for both local and distant cellular communications, as these nanoparticles are able to transfer their cargo into the acceptor cells in autocrine and paracrine fashions [11].

In this study, we evaluated whether the presence of increased levels of miR-222 into melanoma-released EXOs were able to transfer the aggressive behavior of the donors into the acceptor cells. We also evaluated the possible functional role of some miR-222-related molecules carried by purified EXOs in supporting melanoma malignancy.

## Methods

### Cell culture

Human melanoma cell lines were stabilized from surgical specimens obtained from primary or metastatic tumors at Istituto Nazionale Tumori (Milan, Italy). Cell lines were characterized for growth in soft agar and, whenever possible, their metastatic potential was evaluated into athymic nude mice. Early passages cells were obtained from bioptic specimens at Istituto Dermopatico dell’Immacolata (Rome, Italy). All biological materials were obtained with the informed consent of patients and the study was conducted according to the Declaration of Helsinki Principles. An institutional approval for the performed experiments was not required. The cell lines were authenticated according to standard short tandem repeat (STR)-based genotyping. Melanoma cell lines were cultured in Dulbecco’s modified Eagle’s medium (DMEM) (GIBCO by Life Technologies, Paisley, UK) supplemented with 10 % FBS (GIBCO). Cells were incubated at 37 °C and supplemented with 5 % CO_2_ in humidified chamber. When indicated, melanoma cell lines were lentivirally transduced with miR-222 as previously reported [6, 7, 12]. Treatments with NVP-BKM120 (Selleckchem, Houston, TX, USA), a PI3K specific pan inhibitor, were performed at doses ranging between 1 and 5 µM in synchronized or not synchronized melanoma cells, in presence of 5 or 10 % FBS previously deprived of endogenous microvesicles by ultracentrifugation.

### Exosome isolation and tracking analysis

**Fig. 1:**
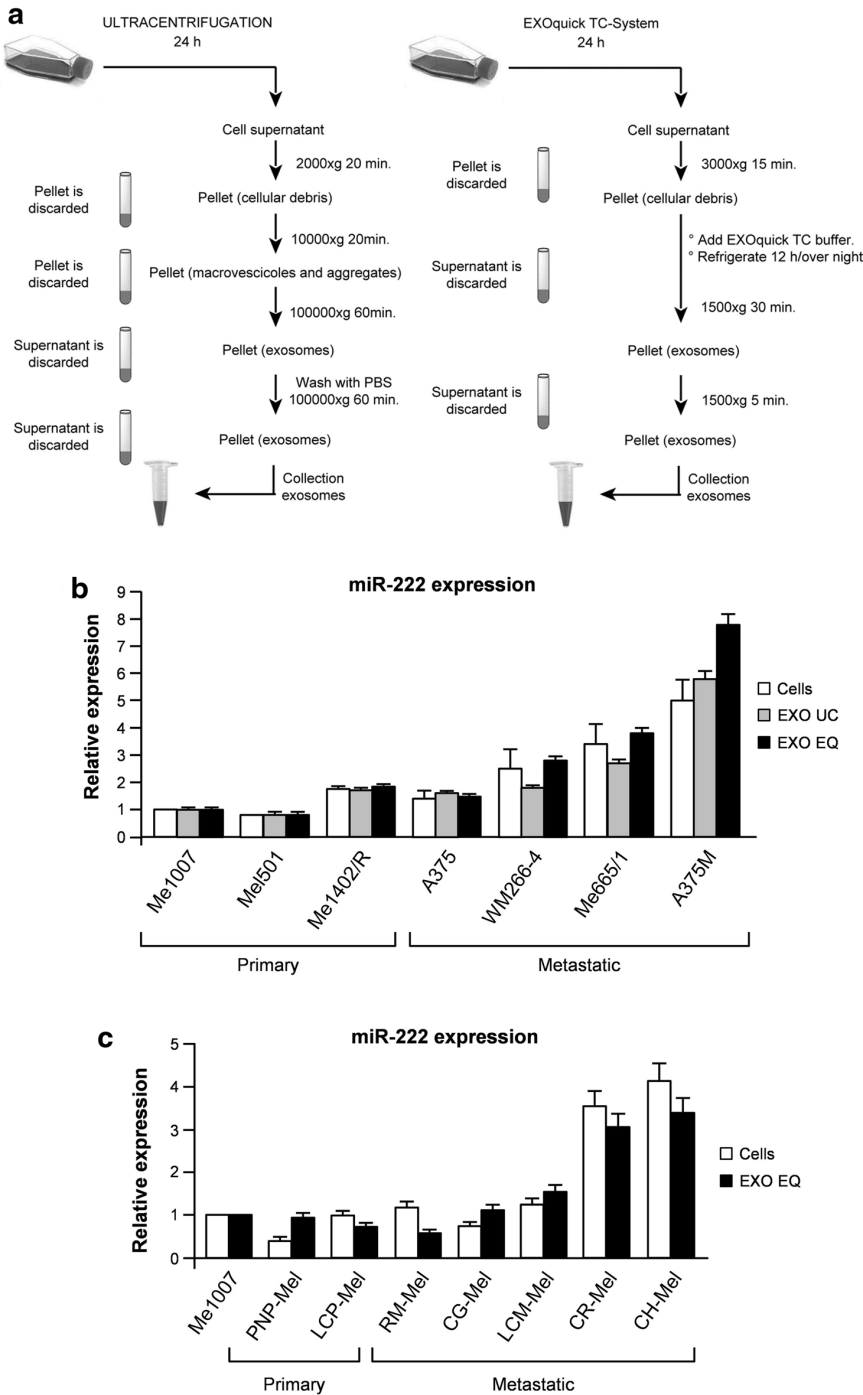
Exosomes (EXOs) purification and miR-222 expression analysis in melanoma. **a** Flow chart outlining the main steps of exosome isolation from cell culture supernatants. EXOs were purified from 24 h cell culture media by ultracentrifugation (UC) (*left panel*) or Exoquick-TC (EQ) (*right panel*) methods. miR-222 levels were compared by qRT-PCR in **b** stabilized melanoma cell lines and corresponding EXOs either UC (EXO UC) or EQ (EXO EQ) purified and **c** in early passages melanoma cell lines and corresponding EXOs. Me1007 primary melanoma cell line was used as an internal control to compare the two groups. Columns, mean ± SD of at least three independent experiments

EXOs were isolated from 24 h cell culture media by ultracentrifugation (UC) or Exoquick-TC (EQ) (System Biosciences, Mountain View, CA, USA) methods according to standards procedures or manufacturer’s instruction, with minor modifications (Fig. 1a). For exosome purification, serum was depleted of bovine EXOs by ultracentrifugation at 100,000×*g* for 6 h, followed by passing it through 0.2 µm filter prior to use. The protein concentration of EXOs was determined using a protein assay kit (Bio-Rad, Hercules, CA, USA) and in some cases the number and size of EXOs were directly tracked using the Nanosight NS300 system (Nanosight™ technology, Malvern, UK), configured with a 488 nm laser and a high-sensitivity sCMOS camera. Videos were collected and analyzed using the NTA software (version 3.0). For each sample, multiple videos of 60 s duration were recorded generating replicate histograms that were averaged.

### Exosome labeling and internalization

Cells were labeled by including into the culture medium (DMEM supplemented with 0.3 % FBS-UC) the fatty acid molecule BODIPY^®^ FL C16. After 5 h of incubation, the dye in excess was washed out and cell culture media containing EXOs (with fluorescent phospholipids incorporated into membranes) were recovered. Thirty micrograms of EXOs (purified with EQ methods) were co-cultured with 2 × 10^4^ recipient cells grown in eight-well chamber slides (IBIDI, Martinried, Germany). After 2–3 h of incubation, cells were fixed in 4 % w/v paraformaldehyde (Sigma–Aldrich) for 10 min. Next, cells were stained by Alexa Fluor 647 conjugate phalloidin (Immunological Sciences, Rome, Italy) and nuclei by Hoechst 333258 dye (Sigma–Aldrich). Exosomal and cellular staining were analyzed by Olympus FV-1000 laser-scanning confocal microscopy.

### In vitro experimental model of fusion and functional assays

The same amounts of EXO/Tween or EXO/miR-222 purified EXOs, recovered from conditioned media of melanoma cells transduced either with Tween empty vector or miR-222, were incubated with recipient cells for 30 min at 37 °C before performing expression studies and functional assays. Vesicle preparations were used immediately after isolation. Invasion and chemotaxis studies were performed according to standard procedures [6]. For tube formation assays, melanoma cells after being fused with EXOs were seeded into culture slide wells coated with 100 mg/cm^2^ of Matrigel growth factor reduced (Becton–Dickinson, Bedford, MA, USA). Tube-like formations defined as ≥2 cells forming elongated structures were counted after 24–48 h of incubation by microscope (JULI microscopy, Twin Helix, MI, Italy) at 10 × magnification from four different fields for each condition. Tube formation was analyzed manually [13] and by the Image J software. Experiments were conducted at least three times.

### MiR-221 and miR-222 silencing by antagomir treatment

Chemically modified antisense oligonucleotides (antagomir or αmiR) were used to inhibit miR expression [6]. The sequences of αmiR-221 and αmiR-222 used are: 5′P-GAAACCCAGCAGACAAUGUAGCU-3′-Chl and 5′P-GAGACCCAGUAGCCAGAUGUAGCU-3′-Chl, respectively; all the bases were 2′OMe modified. Antagomir oligonucleotides, were transfected at 200 nmol/L by using Lipofectamine 2000 (Invitrogen), according to the manufacturer’s procedures. As a control, an unrelated antagomir, specifically the antagomir targeting miR-133a that is not expressed in melanomas (αmiR-133) was transfected as well. EXOs were purified from conditioned media 24 h after transfections.

### Cell cycle analysis

Cell cycle analysis was performed in synchronized or not synchronized melanoma cells. In the first case cells were synchronized by the addition of Hydroxyurea (HU), final concentration 2 mM, per 16 h. Cultures were then washed and medium replaced. From this point, considered as t = 0, cells were monitored while they proceed along the cell cycle after specific treatments (i.e., EXO internalization or BKM120 supplementation). In not synchronized experiments, cells were seeded at roughly 60–70 % confluence and treated as indicated in DMEM supplemented with 5 % FBS in triplicate. Cells were collected, washed in PBS, and suspended in propidium iodide (PI) staining buffer (PBS containing 1 % Triton X-100, 50 mg/ml PI and 50 mg/ml RNase). Cells were then incubated for 30 min (37 °C) and DNA content measured by flow cytometry using a BD FACS Canto cytometer (BD Biosciences, CA, USA).

### Western blot

Western blot analysis was performed according to standard procedures. Exosome samples were lysed in buffer (0.5 % Triton; 300 mM NaCl; 50 mM TrisNaCl) supplemented with protease inhibitor cocktail. Antibodies listed below were used in accordance to the manufacturer’s instructions: CD63 (SBI System Biosciences, Mountain View, CA, USA), RAB5B, TSG101, HSP90 and CycD1 (Santa Cruz Biotechnology Dallas, TX; USA), LAMP2 and CAV-1 (BD Biosciences, CA, USA), RAB27A (Abnova, Taipei City, Taiwan), p85β (Abcam, Cambridge, MA, USA), AKT, ph-AKT^Ser473^ and p27Kip1 (Cell Signaling, Beverly, MA, USA). β-ACTIN (Oncogene Research, La Jolla, CA, USA) was used as a loading control and subsequent quantification. The expression levels were evaluated by the AlphaView (Protein-simple, CA, USA) or Image Quant Software (Uppsala, Sweden).

### RNA preparation and qRT-PCR

RNA was isolated from cell lines and EXOs using the “Total RNA Purification micro Kit” (NorgenBioteK Corp, Canada) according to the manufacturer’s protocol. In the first setting of experiment, to avoid any possible contamination of external RNAs, before exosome purification conditioned media were pre-treated with RNase (Roche, Nutley, NJ, USA) for 10 min at 37 °C. Real time quantification (qRT-PCR) of miR-222 (#000525), p27Kip1 (#Hs00153277_m1), FGF2 (#Hs00266645_m1), VEGFA (#Hs000900055_m1), ITGβ3 (#Hs00173978_m1) and Bcl-2 (#Hs00153350_m1) were performed according to the TaqMan technology (Applied Biosystems, Foster City, CA, USA). MiR-16 (#000391), RNU6B (#001093) and GAPDH (4326317E) were used as internal controls. The expression profile of Human Tumor metastasis genes was performed using the TaqMan Array 96-Well Plate (#4414229) (Applied Biosystems, Foster City, CA, USA).

### Statistical analysis

Differences were statistically evaluated using Student’s t test. *p* < 0.05 was defined as statistically significant. ANOVA analysis was performed using GraphPad version 4.0 for Windows (GraphPad Software, San Diego, CA) followed by Student–Newman–Keuls post hoc test when appropriate.

## Results

### Characterization of melanoma purified EXOs

MiR-222 overexpression is involved in the poor prognosis of several tumors where it was demonstrated to promote cell growth, migration and invasion and to inhibit apoptosis [14, 15]. In addition circulating miR-222 has been proposed as diagnostic and prognostic marker [16].

In view of the tumorigenic role played by miR-222 in melanoma, we evaluated the exosome capability of carrying miR-222 and its associated properties. For this purpose EXOs were purified, either by ultracentrifugation (UC) or Exoquick-TC (EQ) methods, from conditioned media of stabilized and early passage melanoma cell lines at different stages of progression (Fig. 1a). According to the general trend of miR-222 enhancement associated with melanoma advancement [6], qRT-PCR analysis revealed that EXOs, either UC- or EQ-purified, released by metastatic cells contained higher levels of miR-222 in comparison with primary melanomas (Fig. 1b). More important, to rule out any possible artifact due to cell cultures, this expression pattern was confirmed in melanoma cell lines analyzed at early times after surgical excision and in the corresponding released EXOs (Fig. 1c).

**Fig. 2:**
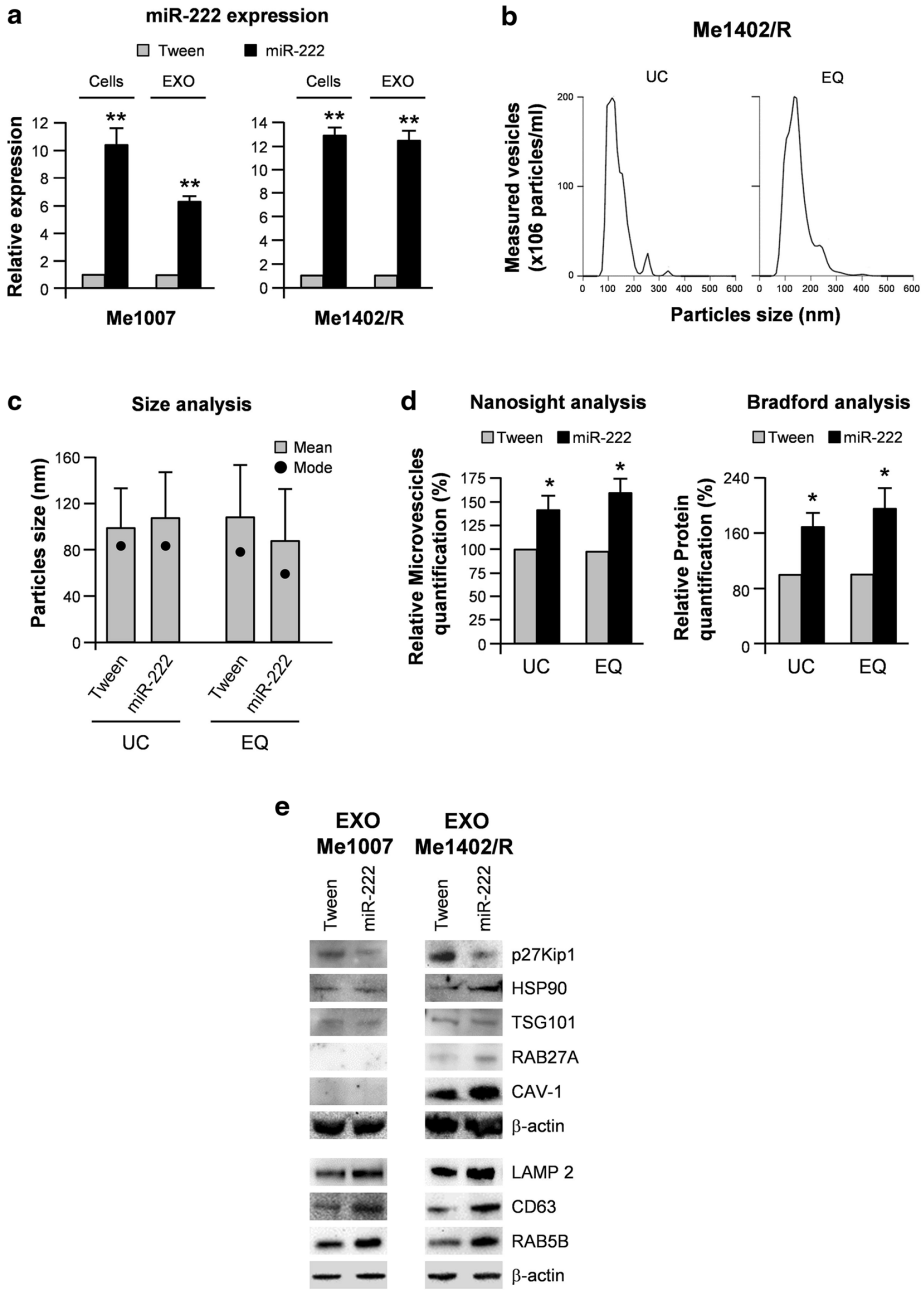
Characterization of melanoma exosomes. **a** Representative qRT-PCR in miR-222- and empty vector-transduced melanoma cells and corresponding exosomes (EXO EQ). Relative miR expression levels were normalized on miR-16 (for EXOs) or RNU6B (for cells). **b**, **c** Size distribution of Me1402/R UC and EQ purified vesicles analyzed by the Nanosight™ technology. *Graph bars* represent the mean size of the particles ±SD, whereas the mode, shown as a *single point* overlying the *graphs*, indicates the most often occurring dimension. **d** The relative amounts of melanoma-derived EXOs were assessed by using the Nanosight™ technology (*left*) or by the Bradford assay for protein quantization (*right*). **e** WB analysis of specific ‘‘exosome-enriched’’ proteins in EXOs purified from Me1007 and Me1402/R melanomas by the EQ method. β-Actin was utilized as internal loading control for each experiment. Columns, mean ± SD of at least three independent experiments. *p < 0.05; **p < 0.01

To investigate the functionality of miR-222 in exosome-mediated tumorigenesis, we lentivirally transduced the Tween control vector or miR-222 in two melanoma cell lines, Me1007 and Me1402/R, early primary and recurrence of primary melanoma, selected in view of their low endogenous levels of this miR (Fig. 1b) [6]. qRT-PCR analysis confirmed the significant relative up-regulation of miR-222 in miR-transduced Me1007 and Me1402/R (10-fold and 12-fold, respectively) in comparison with levels detected in vector-transduced melanoma cell lines (Fig. 2a). Notably, similar increments of miR-222 were detected in the corresponding EXOs (6-fold for EXOs secreted by Me1007 and 12-fold for those released by Me1402/R) (Fig. 2a).

Melanoma-derived vesicles were then characterized for their size distribution by using the Nanosight™ technology. Results showed similar profiles for UC- and EQ-derived EXOs, with nanoparticles ranging between 70 and 140 nm, with a mean value of 100 nm, in both control and miR-222 overexpressing cells (Fig. 2b, c and data not shown). The amount of melanoma-released EXOs was also assessed by using either the Nanosight™ technology or the Bradford assay for protein quantitation. In both cases results revealed that miR-222-transduced melanomas secreted a significantly higher number of EXOs compared with control cells, possibly suggesting a role for miR-222 in the EXO releasing process (Fig. 2d).

The protein content of these EXOs analyzed by western blot showed a miR-222-dependent enrichment for proteins commonly utilized as exosomal markers, such as LAMP2, HSP90, CD63 and RAB5B, (Fig. 2e). In addition, besides confirming miR-222 overexpression into the exosomal cargo (Fig. 2a), we observed reduced levels of p27Kip1, a negative regulator of cell cycle previously demonstrated as a direct target of this miR [6]. These results suggested that miR-222 was somehow functional in the vesicular fraction (Fig. 2e). Accordingly, in Me1402/R we detected a miR-222-dependent increase of CAV-1 and RAB27A, proteins already described for their involvement in exosome uptakes and secretory pathways [17–19]. Although CAV-1 and RAB27A are expressed in Me1007 cells, we were unable to detect them in the corresponding EXOs, likely because of their low levels. As internal loading controls, we utilized TSG101 and β-ACTIN which appeared constantly expressed (Fig. 2e).

### In vitro functional studies on EXO/miR-222

To determine whether the EXO/miR-222 and their cargo could be taken up by recipient cells, we utilized an in vitro experimental model where EXOs isolated from donor cells were incubated with control melanoma cell lines. Specifically the same amounts of EXOs, purified either from miR-222- or from empty vector-transduced melanoma cells, were incubated with Me1007 or Me1402/R acceptor cells.

**Fig. 3:**
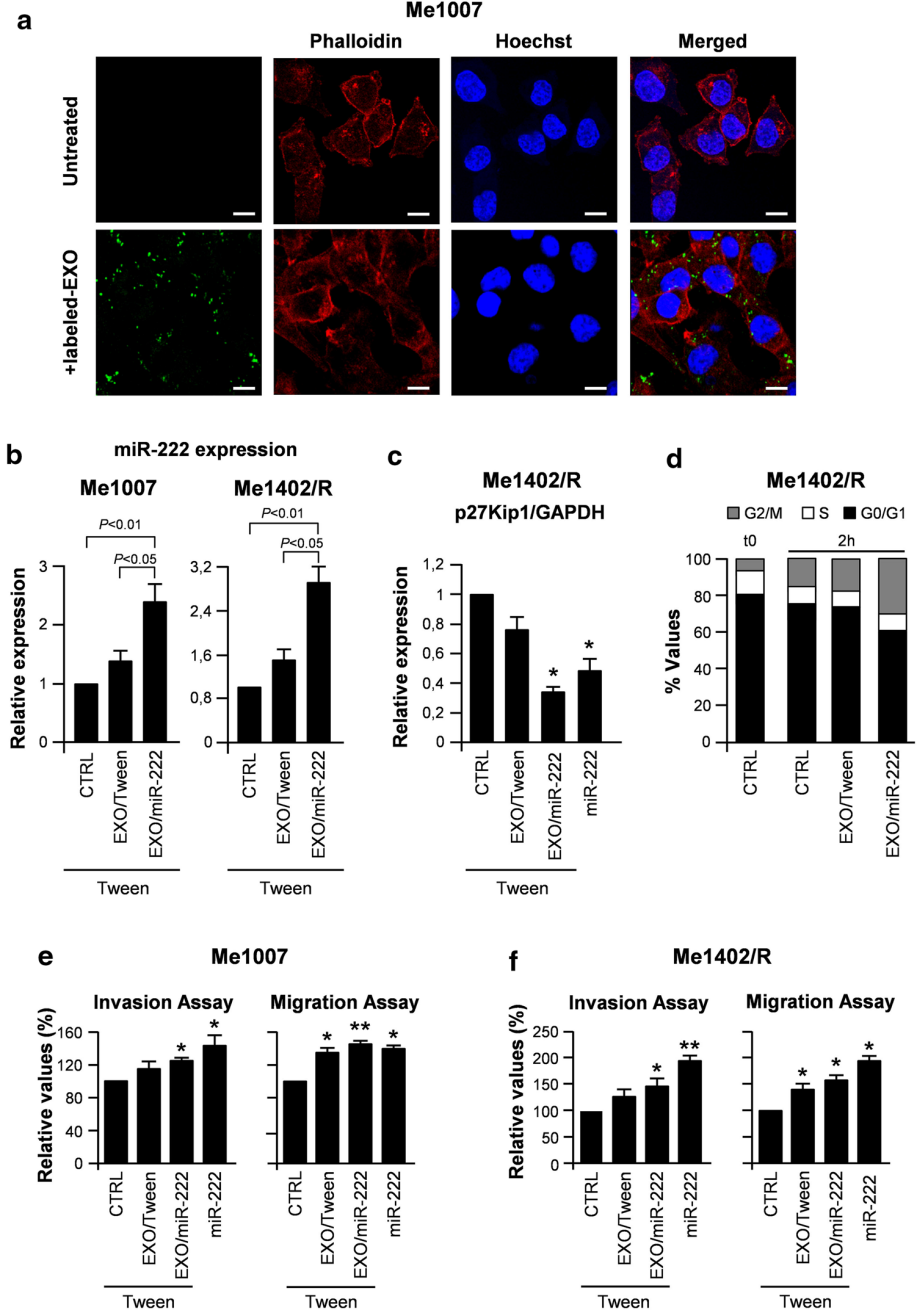
Evaluation of exosome uptake by recipient cells. **a** Confocal microscopy visualization of untreated (*top*) and exosome fused (*bottom*) Me1007 cell line. Me1007/miR-222 donor cells were labeled with the fluorescent BODIPY^®^ FL C16 fatty acid molecule, which being incorporated into the EXO membranes allowed visualizing these vesicles. Me1007 recipient cells were stained for phalloidin (Alexa Fluor 647-red) and nuclei counterstained with Hoechst. Bodipy C16-labelled exosomes appear as internalized *green dots*. *Scale bar* 10 μm. **b** The uptake of miR-222-containing exosomes by the acceptor Me1007 and Me1402/R melanomas was quantified by specific qRT-PCR (miR-222/RNU6B). Data are cumulative of three independent experiments. Differences in miR-222 expression were evaluated using analysis of variance (ANOVA) followed by a Newman–Keuls post hoc test. Significance was accepted when the p value was <0.05. **c** The downregulation of p27Kip1, a direct target of miR-222, by EXO/miR-222 was evaluated by qRT-PCR. **d** Cell cycle analysis, showing the miR-222-dependent early onset of DNA synthesis, was performed on synchronized cells 2 h after exosome internalization. **e**, **f** EXO-dependent effects on invasion (*left panels*) and chemotaxis (*right panels*) in Me1007 and Me1402/R melanoma cell lines. MiR-222-transduced cells were included as a positive control. Data are representative of two independent experiments. *p < 0.05; **p < 0.01

To visualize the actual internalization of the vesicles and the effectiveness of miR-222 transfer from donor into acceptor cells, Me1007/miR-222 cells were stained with the fluorescent fatty acid molecule BODIPY^®^ FL C16 which, being incorporated into membrane phospholipids, made possible EXOs’ labeling and in turn visualization. Me1007 cells and miR-222-containing labeled EXOs were then incubated for 2 h at 37 °C before confocal microscopy evaluation. Control untreated and exosome fused cells were stained by phalloidin (Alexa Fluor 647, red) and by the nuclear Hoechst dye (blue). The internalization of EXOs was evidenced by a green fluorescent punctuate signal inside the cytoplasm of Me1007 recipient cells (Fig. 3a). Similar results were obtained in Me1402/R cellular model (data not shown).

Above all the horizontal transfer was confirmed by specific qRT-PCR for miR-222. Indeed the uptake of miR-222-containing EXOs by the acceptor melanoma cells produced 3-fold increase of miR-222 respect to its basal expression in Me1402/R/Tween cells and 1.5-fold respect to the homologous control fusion with EXO/Tween (Fig. 3b right). Similar results were obtained in Me1007 melanoma cells (2.5-fold increase of miR-222 respect to its basal cellular expression and 1.3-fold respect to the EXO/Tween control Fig. 3b left). Of note was the downregulation of p27Kip1 consequent to EXO/miR-222 internalization comparable to that obtained by miR-222 lentiviral-induced overexpression (Fig. 3c). In addition the horizontal transfer was confirmed by changes of some other relevant molecules. As suggested by their higher expression in EXO/miR-222 respect to EXO/Tween (Fig. 2e), we observed the exosome-related capability to convey vesicle-markers possibly associated with tumorigenesis, such as CD63, CAV-1, RAB5B and RAB27A, into the acceptor cells (Additional file 1: Fig. S1).

**Fig. 4:**
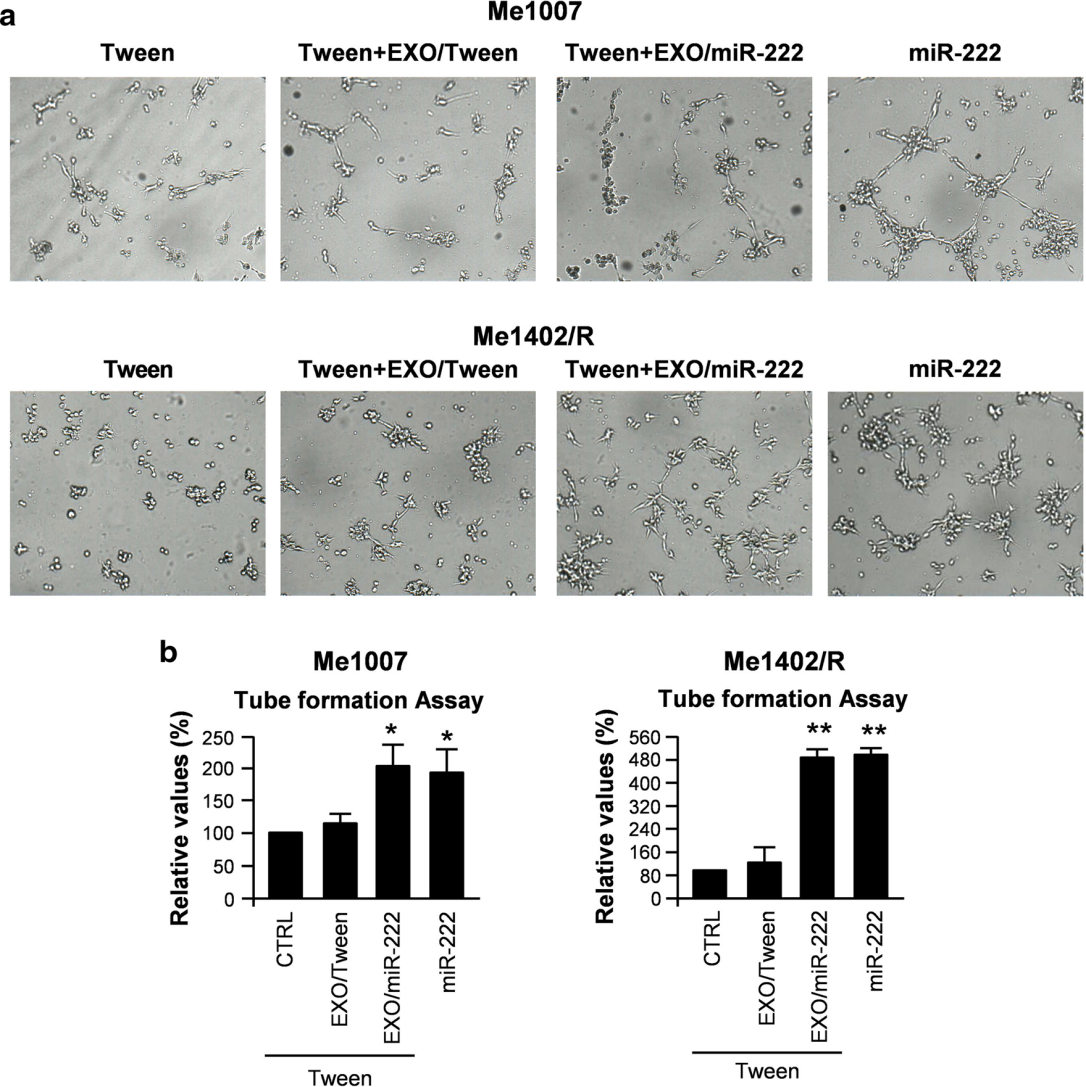
In vitro functional studies in Me1007 and Me1402/R. **a** Morphological and **b** quantitative analyses of tube formation and length in Tween- and miR-222-transduced melanoma cells compared with EXO/Tween or EXO/miR-222 fused Me1007 or Me1402/R cells. Purified exosomes were incubated with recipient cells for 30 min at 37 °C before performing functional assays. Tube formation was analyzed 20 h after exosome internalization. Data are representative of three independent experiments. *p < 0.05; **p < 0.01

Basing on these results, we searched for any tumorigenic effect possibly associated with EXO/miR-222. At first, and in view of p27Kip1 decrease, we evaluated the cell cycle rate possibly modulated as a consequence of EXO/miR-222 internalization. As already shown for melanoma cells overexpressing miR-221 or miR-222, where previous analyses revealed an early onset of DNA synthesis paralleled by G0/G1 reduction [6], we detected an increased proliferative rate when Me1402/R cells were incubated with EXOs enriched for miR-222. Specifically, 2 h after serum stimulation cell cycle evaluation showed either in control or in EXO/Tween treated: 75–78 % of the cells in G0/G1, 7–8 % in S and 14–18 % in G2/M. Values were modified by EXO/miR-222 uptake up to 62 % G0/G1, 8 % S and 30 % G2/M (Fig. 3d). Also by using a Boyden chamber assay we observed a small, but significant EXO/miR-222-dependent induction of the invasive and chemotactic capabilities (Fig. 3e, f). More evident was the different capability in the vessel-like process formation, which partly mimics melanoma aggressiveness [13]. Indeed the EXO/miR-222 fusion enhanced the capability of forming vascular-like structures by Me1007 and Me1402/R (Fig. 4a, b).

**Fig. 5:**
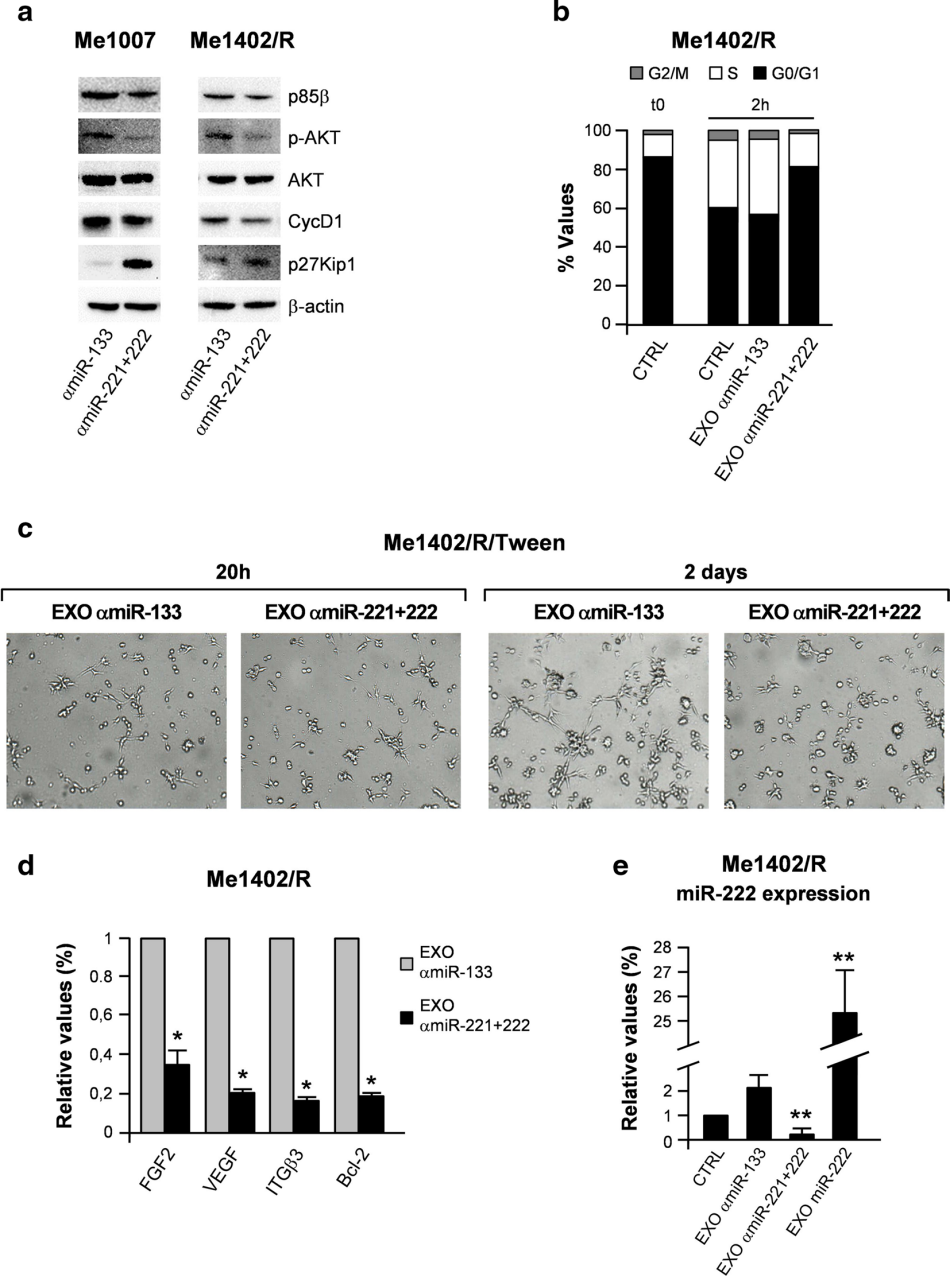
Effects on melanoma tumorigenesis of antagomir-221&222 carried by EXOs. Melanoma cells treated with control antagomir-133 (αmiR-133)- or antagomir-221&222 (αmiR-221 + 222)-EXOs were compared for **a** modulation of protein expression by western blot, **b** cell cycle rate, **c** tube-like formation capability evaluated at 20 h and 2 days after EXO internalization. qRT-PCR evaluation of **d** miR-222 related molecules and **e** miR-222 itself to confirm its inhibition. Relative miR expression levels were normalized on RNU6B. Data are representative of two independent experiments. *p < 0.05; **p < 0.01. β-Actin and GAPDH were utilized as internal controls

The reverse effects were obtained with EXOs recovered after inhibition of endogenous miR-221 and miR-222 by antagomir transfections. The choice of abrogating both miRs derive from their possible redundant roles based on the high number of shared target genes [6]. Previous studies showed reduced cell proliferation and slight decrease in invasion and migration abilities in melanoma cell lines transfected with these highly stable oligomers showed [6]. Accordingly, we observed the antago-dependent reduction of the PI3K/AKT and cyclin D1 (CycD1) axis, together with the upregulation of p27Kip1 (Fig. 5a). In line with miR-221&222 abrogation, the antagomir-carrying EXOs were able to reduce the cell cycle rate of melanoma acceptor cells (EXO/αmiR-221&222 treatment: 82 % of the cells in G0/G1, 17 % in S, 1 % in G2/M vs control or EXO/Tween treatment: 57–62 % in G0/G1, 34–39 % in S, 4 % in G2/M) (Fig. 5b). Yet again more evident differences were detected in the formation of vascular-like structures resulting reduced and less organized by the αmiR-221&222/EXO internalization (Fig. 5c). In line with these functional effects, we detected the regulation of some key factors involved in cell growth, apoptosis and tube formation, in particular reduction of Bcl2, ITGβ3, FGF2 and VEGF (Fig. 5d). The specificity of miR-222 down-regulation was confirmed by qRT-PCR (Fig. 5e).

### Activation of the PI3K/AKT pathway by EXO-miR-222

**Fig. 6:**
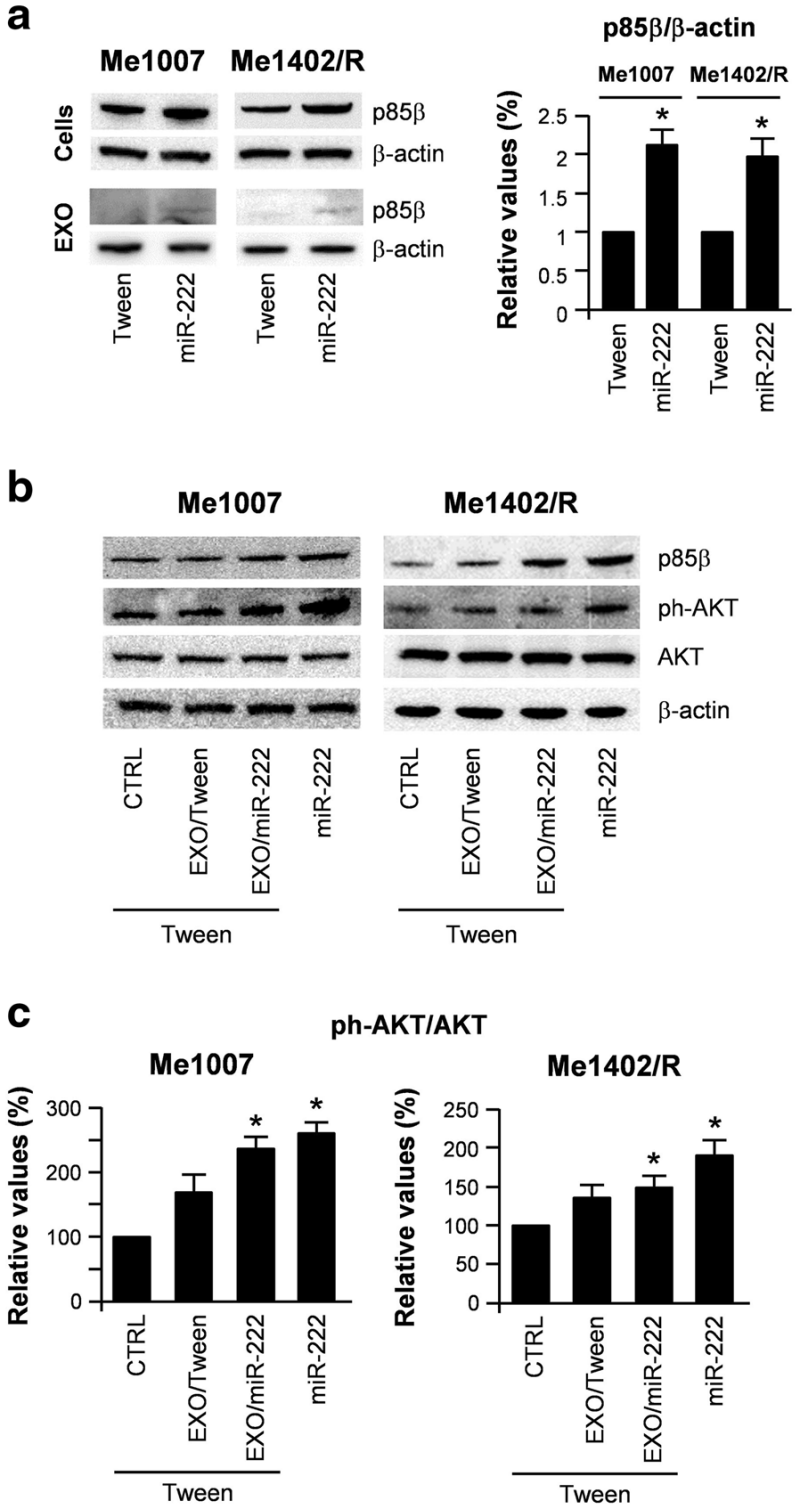
WB analysis of proteins modulated by EXO/miR-222 in Me1007 and Me1402/R melanomas. **a** Western blot analysis of PI3K/p85β subunit in Tween control vs miR-222-transduced cells and corresponding EXOs (*left panel*) and relative densitometric quantification (*right panel*). **b** Western blot analysis of PI3K/AKT related proteins after fusion of either EXO/Tween or EXO/miR-222 with Tween-transduced melanomas. β-Actin was utilized as internal loading control. **c** Quantification of ph-AKT^Ser473^/Akt ratios. Mean ± SD of three independent experiments *p < 0.05

As the PI3K/AKT pathway is frequently deregulated in cancer, the main components of this axis represent attractive candidates for targeting. A growing body of evidence has shown that MAPK and PI3K signaling play major roles in melanoma development and progression [20]. In addition the expression of miR-221 and miR-222 has been reported to be under the positive control of the RAS/MAPK [21, 22] and upstream to PI3K/AKT signaling in different cellular models [23, 24]. As miR-221&222 deregulation has been associated with these tumorigenic pathways and circulating miR-222 was suggested as a possible tumor biomarker [25–28], we evaluated whether EXOs secreted by miR-222-overexpressing cells might be able to induce an oncogenic program through the horizontal transfer of miR-222 itself as well as of related molecules. Expression studies confirmed a miR-222-dependent upregulation of the PI3K/p85β subunit in both melanoma cells and EXOs (Fig. 6a) and the reverse results in antagomiR-221&222 treated cells (Fig. 5a). We then investigated whether the transfer of EXO/miR-222 might be sufficient to modulate the PI3K/AKT pathway after internalization into the recipient cells. Western blot analysis showed the increase of PI3K/p85β and ph-AKT^Ser473^, key molecules involved in this signaling, up to levels similar to those of miR-222-transduced cells (Fig. 6b). Densitometric quantifications of total AKT and its Ser^473^ phosphorylated fraction confirmed the increased ph-AKT^Ser473^/total AKT ratios associated with miR-222 (Fig. 6c).

**Fig. 7:**
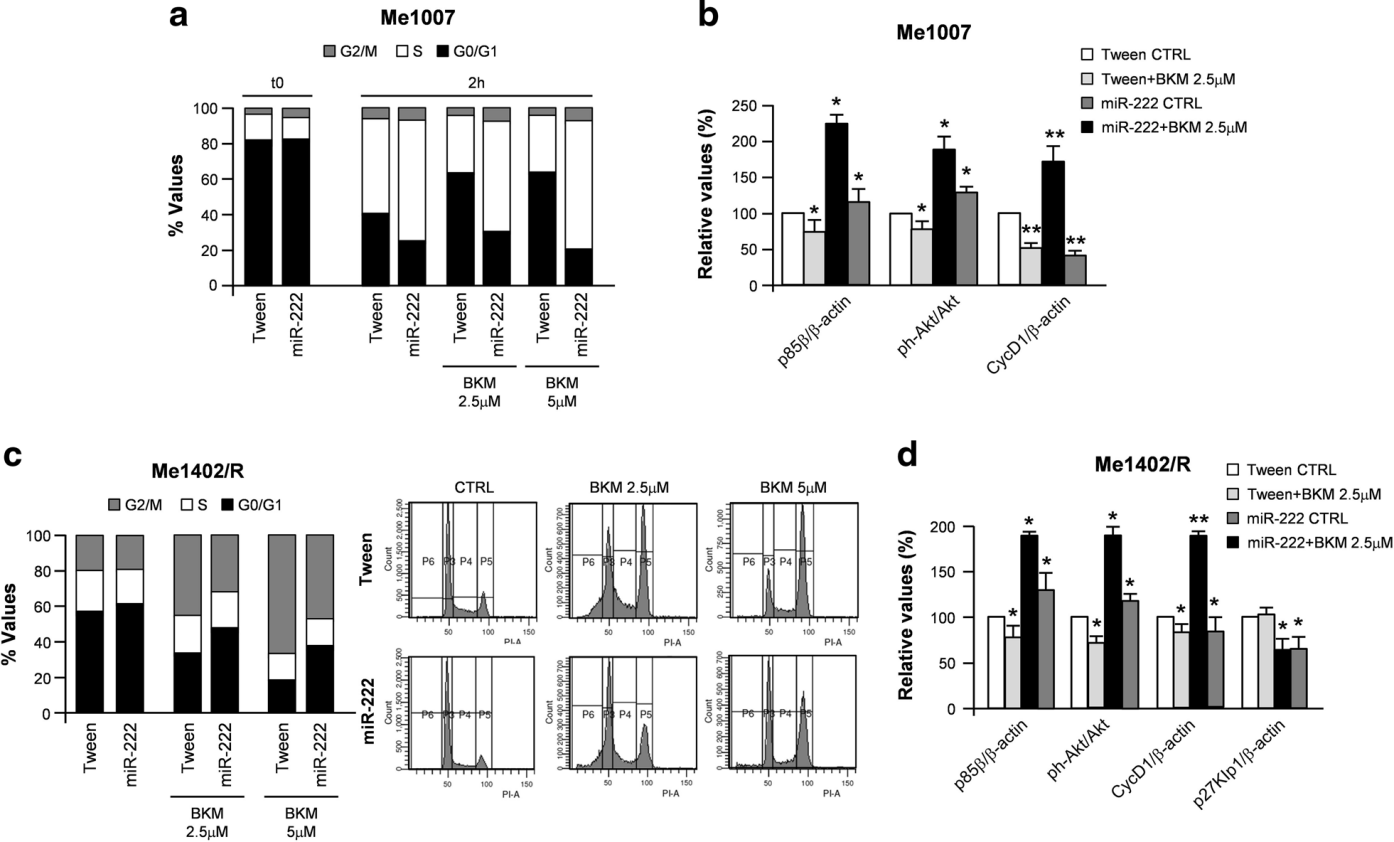
Effects of miR-222 overexpression on the inhibition of PI3K/AKT axis. Cell cycle analysis of miR-222-transduced vs control cells treated with 2.5 and 5 µM of BKM 120 in **a** synchronized Me1007 and **c** proliferating Me1402/R. **b** and **d** Relative densitometric quantification of western blot performed in the same cells. β-Actin was utilized as internal loading control. Data are representative of two independent experiments. *p < 0.05; **p < 0.01

To assess the possible differential significance of PI3K blockade in control vs miR-222-transduced cell lines, we treated either synchronized or not synchronized melanoma cells with NVP-BKM120, a potent class I PI3K pure pan inhibitor, at doses ranging between 2.5 and 5 µM. Results were evaluated on cell cycle rates. In both treatments, miR-222 overexpression seemed to interfere with BKM120-dependent effects. In hydroxyurea-blocked cells, the 2 h cell cycle determination showed the miR-222-dependent earlier onset of DNA synthesis in both untreated and BKM120-treated Me1007/miR-222 compared with control vector transduced cells (Fig. 7a). When the effects of BKM120 were evaluated on proliferating melanoma cells, miR-222 seemed to interfere with the BKM120-dependent block in the G2/M phase (Fig. 7c). Western blot analyses and densitometric quantitations confirmed ph-AKT^Ser473^ and PI3K/p85β downregulation (Fig. 7b, d and Additional file 2: Fig. S2).

To further dissect the role of miR-222 in the exosome cargo of Me1402/R melanoma, we compared the expression profiles of EXO/miR-222 and EXO/Tween by using the TaqMan array plate for human tumor metastasis genes (Additional file 3: Fig. S3A). Although current reports have shown that a fraction of the RNAs present in EXOs could be somewhat degraded, the presence of some poly-adenylated RNAs was also reported [29, 30]. As possibly expected in view of the low amounts of RNA recovered from EXOs, qRT-PCR analyses revealed that the majority of genes exhibited Ct values higher than 35. Even so, in agreement with miR-222 tumor promoting function, we observed the up-regulation of a number of genes involved in melanoma progression. Among them we found the miR-222-based upregulation of VEGF and FGF2 well known to play major roles in melanoma cell growth and tumor angiogenesis according to autocrine and paracrine functions [31, 32]. Interestingly, some other factors, as the MGAT5, MCAM and TGFβ1, cooperating in the induction of prometastatic phenotypes in melanoma [33, 34], were induced by miR-222. Finally, among the few genes downregulated in EXO/miR-222 we detected MTA1 and MTA2, nuclear receptor coregulators overexpressed in human cancers, but reported to play a dual role being either corepressors or coactivators [35]. The accuracy of these microarray results was validated by qRT-PCR of FGF2 and VEGF genes (Additional file 3: Fig. S3B).

## Discussion

MiRs are non-coding RNAs regulating gene expression mostly at post-transcriptional level [36]. Growing evidences are showing their functional involvement in all the main biological processes, including cancer development and progression, where they can act either as oncogenes or tumor suppressors [3]. Besides their cellular association, recent data demonstrated miRs to be secreted in the extracellular compartments as free molecules or carried by microvesicles, thus transferring information at local and distant sites [30].

Several groups have focused their attention on miR-221 and miR-222 reported to drive the oncogenesis of many types of malignancies by directly repressing a number of tumor suppressor genes and activating oncogenic pathways [14]. Focusing on melanoma, we previously showed that miR-221&222 play a dynamic role regulating proliferation and progression repressing several antineoplastic targets (e.g., p27Kip1/CDKN1B, c-KIT receptor and c-FOS) [6, 7].

Here we show that miR-222, being part of melanoma exosomal cargo, can be transferred between cells resulting per se able to promote tumorigenesis through the activation of several molecules, including the PI3K/AKT pathway.

Since a definite consensus on the ‘‘correct’’ technique to isolate EXOs is still lacking [37], we utilized and compared ultracentrifugation and Exoquick alternative methods. Purified EXOs, analyzed by NanoSight and/or western blot technologies, showed the same distribution as well as similar enrichment for some vesicular proteins, as LAMP2, CD63, RAB5B, TSG101, HSP90 and β-ACTIN (Fig. 2). In agreement with the general gradual increase of miR-222 associated with melanoma progression, either in stabilized or early passage cell lines, we detected the same expression pattern in the corresponding EXOs (Fig. 1). Moreover a significant accumulation of this miR was detectable in EXOs purified from primary melanoma cell lines (Me1007 and Me1402/R) enforced to express miR-222 (Fig. 2). Indeed the mature sequence of miR-222 contains two short sequence motifs reported to function as exosomal packaging signals or miR intracellular retaining [38].

Previous studies already provided evidences of exosome transfer and content deliver from donors to recipient cells [30, 39]. Utilizing an experimental model, we got higher invasive and chemotactic capabilities and, even more evident, vessel-like process formation associated with EXO/miR-222 internalization into primary melanomas, indicating increased melanoma malignancy (Figs. 3, 4) [13]. The reverse effects obtained after internalization of EXOαmir-221&222 supported our conclusions (Fig. 5). An analogous EXO-based increase of malignancy was described for miR-10b in breast cancer [9, 40] where miR-10b was associated with the risk of relapse [41, 42].

Trying to dissect the downstream pathways regulated by miR-222, we demonstrated, either in melanoma cells or secreted EXOs, the miR-222-dependent induction of the PI3K/AKT pathway, associated with the expected downregulation of miR-222 direct target p27Kip1. As no significant differences were detected in the cell-cycle rates of miR-222-overexpressing cells treated or not with the AKT inhibitor BKM120 (Fig. 7a), we considered the capability of miR-222 to overcome the inhibition of the PI3K/AKT pathway, on one side considering the frequent constitutive activation of the MAPK axis, on the other the number of genes inhibiting proliferation, inducing apoptosis or generally playing tumor suppressor functions, known to be directly targeted by miR-222 [14]. In addition PI3K/p85β was identified as a direct target of miR-126, that we recently demonstrated as part of a cross regulatory circuitry linking the oncomiR-221&222 with the tumor suppressors miR-126&126*. During melanoma progression, the expression levels of these two couples of miRs functionally move from miR-126&126* toward miR-221&222 under the regulation of the transcription factor AP2α [43]. In line with their opposite roles, protein analyses confirmed that several miR-126&126* targeted genes were induced by miR-221&222, including AKT and PI3K/p85β.

According to the reported association between the number of released EXOs and tumor malignancy [19], miR-222 seemed also to augment the amount of secreted EXOs per cell. In agreement with their functional roles recently associated with cancer, some exosomal markers resulted upregulated by miR-222 (Fig. 2). Among them, RAB GTPases were implicated in membrane trafficking and exosome secretion in melanoma. Specifically RAB5B and RAB27 were shown to increase the release and transfer capability of the microvesicles being involved in tumor metastatization including melanoma [19, 44–46]. Also the tetraspanin CD63, a well known marker of microvesicles, was associated with prometastatic pathways [47] and evidenced together with Cav1 on plasma EXOs of melanoma patients [18]. Previous studies linked Cav1 overexpression with melanoma malignancy showing that its secreted amounts loaded in the exosomal cargo were involved in cell migration [17, 48]. Indeed, recent studies supported the notion of the pro-metastatic role of CD63 through β-catenin induction and subsequent increase of ERK phosphorylation and PI3K/AKT pathway activity [47, 49, 50]. Although these increases might suggest the involvement of miR-222 in the EXO releasing process, at present no significant data directly correlate these exosomal markers with miR-222.

The expression profiles obtained by analyzing a panel of tumor metastasis genes further demonstrated the presence of higher levels of tumor promoting genes in EXO/miR-222. Among them we found MGAT5, which in melanoma plays a role during the transition from the vertical growth phase to the metastatic stage, together with its targets MCAM [33], and TGFβ expressed in most malignant melanomas and correlating with poor survival [51]. Last but not least the increased levels of the growth factors VEGF and FGF2 found into the exosome cargo (Additional file 3: Fig. S3A), besides underlying the miR-222 induction of vascular-like structures, suggested the exosome-based transport to explain the unconventional leaderless secretion of FGF2 [52].

## Conclusions

The growing understanding of cancer cell-derived vesicles, exosome-mediated uptake and transfer of the molecular cargos is making more realistic to easily evaluate EXOs in plasma from patients. Our results implicate miR-222, either cell-associated or exosome-transported, as a regulator of melanoma malignancy (Additional file 3: Fig. S3C), suggesting its potential validity as diagnostic and prognostic biomarker. Indeed, as miR-222 was reported to be under the positive control of the RAS/MAPK and upstream to PI3K/AKT signaling, its abrogation might represent a promising therapeutic option.

## Additional files

10.1186/s12967-016-0811-2 Evaluation of exosome-enriched proteins in Me1007 and Me1402/R melanomas. Western blot analysis after fusion of EXO/Tween or EXO/miR-222 on Tween-transduced melanomas. MiR-222-transduced cells were included as positive control and β-actin utilized as an internal loading control.
10.1186/s12967-016-0811-2 Effects of BKM120 treatment. Western blot analysis of PI3K/AKT and cell-cycle-related proteins in miR-222-transduced vs control cells β-actin was utilized as internal loading control. Data are representative of two independent experiments.
10.1186/s12967-016-0811-2 Expression profiling of the exosomal cargo. **A)** Differentially expressed genes obtained by the TaqMan Array Plate for Human Tumor Metastasis genes in EXO/miR-222 vs EXO/Tween samples. **B)** The expression level of some selected genes modulated in TaqMan Array Plate was confirmed by qRT-PCR. **C)** Schematic illustration of pathways regulated by EXO/miR-222 in melanoma.

## Abbreviations

miR: microRNA
qRT-PCR: real time quantification
p27Kip1/CDKN1B: cyclin-dependent kinase inhibitor 1B
c-KIT: v-kit hardyzuckerman 4 feline sarcoma viral oncogene homolog
FOS: v-fos fbj murine osteosarcoma viral oncogene homolog
BODIPY^®^ FL C16: 4,4-Difluoro-5,7-Dimethyl-4-Bora-3a,4a-Diaza-*s*-Indacene-3-Hexadecanoic Acid
TSG101: tumor susceptibility gene 101 protein
CD63: melanoma-associated antigen MLA1
CAV-1: caveolin-1
HSP90: heat shock protein 90
LAMP2: lysosome-associated membrane glycoprotein 2
RAB5B: ras-related protein Rab-5B
p85β: phosphatidylinositol 3-kinase regulatory subunit beta
RAB27A: ras-associated protein
PI3K: phosphatidylinositol 3-kinase
AKT: v-akt murine thymoma viral oncogene homolog 1
VEGFA: vascular endothelial growth factor a
FGF 2: fibroblast growth factor 2
MGAT5: n-acetylglucosaminyltransferase v
TGFβ1: transforming growth factor, beta-1
MTA: metastasis-associated gene
MCAM: melanoma cell adhesion molecule
TIMP1: metalloproteinase inhibitor 1
CycD1: cyclin D1
ITGβ3: integrin β3
Bcl2: B-cell cll/lymphoma 2
