# Supplementary material for: Exosome-mediated transfer of miR-222 is sufficient to increase tumor malignancy in melanoma
Source: J Transl Med. 2016 Feb 24;14:56. doi: 10.1186/s12967-016-0811-2 (PMC4765208; doi:10.1186/s12967-016-0811-2)
Supplement: Supplementary file 1 — 10.1186/s12967-016-0811-2 Evaluation of exosome-enriched proteins in Me1007 and Me1402/R melanomas. Western blot analysis after fusion of EXO/Tween or EXO/miR-222 on Tween-transduced melanomas. MiR-222-transduced cells were included as positive control and β-actin utilized as an internal loading control. [file 12967_2016_811_MOESM1_ESM.pdf]

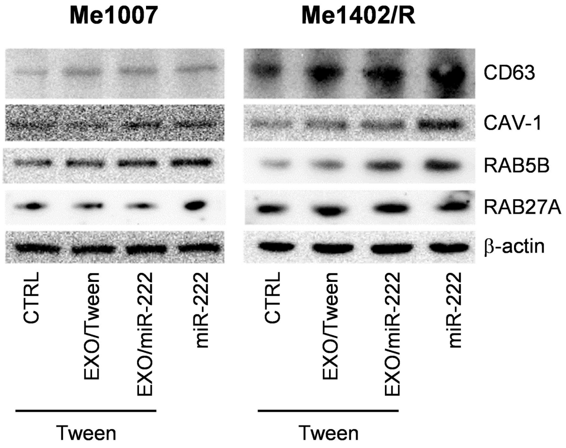

Supplementary Figure 1. Evaluation of exosome-enriched proteins in Me1007 and Me1402/R melanomas. Western blot analysis after fusion of EXO/Tween or EXO/miR-222 on Tween-transduced melanomas. MiR-222-transduced cells were included as positive control and β-actin utilized as an internal loading control.
