# Supplementary material for: Exosome-mediated transfer of miR-222 is sufficient to increase tumor malignancy in melanoma
Source: J Transl Med. 2016 Feb 24;14:56. doi: 10.1186/s12967-016-0811-2 (PMC4765208; doi:10.1186/s12967-016-0811-2)
Supplement: Supplementary file 2 — 10.1186/s12967-016-0811-2 Effects of BKM120 treatment. Western blot analysis of PI3K/AKT and cell-cycle-related proteins in miR-222-transduced vs control cells β-actin was utilized as internal loading control. Data are representative of two independent experiments. [file 12967_2016_811_MOESM2_ESM.pdf]

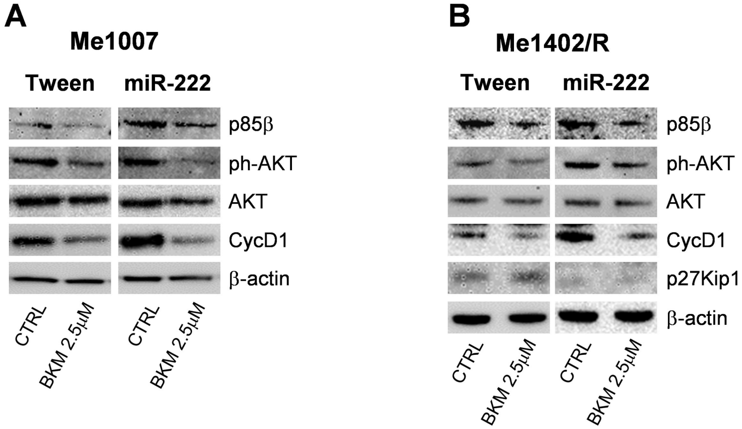

Supplementary Figure 2. Effects of BKM120 treatment. Western blot analysis of PI3K/AKT and cell-cycle –related proteins in miR-222-transduced vs control cells β-actin was utilized as internal loading control. Data are representative of two independent experiments.
