# Supplementary material for: Exosome-mediated transfer of miR-222 is sufficient to increase tumor malignancy in melanoma
Source: J Transl Med. 2016 Feb 24;14:56. doi: 10.1186/s12967-016-0811-2 (PMC4765208; doi:10.1186/s12967-016-0811-2)
Supplement: Supplementary file 3 — 10.1186/s12967-016-0811-2 Expression profiling of the exosomal cargo. A) Differentially expressed genes obtained by the TaqMan Array Plate for Human Tumor Metastasis genes in EXO/miR-222 vs EXO/Tween samples. B) The expression level of some selected genes modulated in TaqMan Array Plate was confirmed by qRT-PCR. C) Schematic illustration of pathways regulated by EXO/miR-222 in melanoma. [file 12967_2016_811_MOESM3_ESM.pdf]

A

Assay ID	Gene Symbol	ΔCt	EXOmiR-222 vs EXOtween
Hs00900054_m1	VEGFA	-4,1	up
Hs00266645_m1	FGF2	-3,2	up
Hs00159136_m1	MGAT5	-2,7	up
Hs99999918_m1	TGFB1	-2,6	up
Hs00261399_m1	KISS1R	-2,6	up
Hs00183425_m1	SMAD2	-2,5	up
Hs00174838_m1	MCAM	-2,5	up
Hs00738978_m1	NF2	-2,2	up
Hs00170192_m1	PNN	-2,2	up
Hs00171558_m1	TIMP1	-2,2	up
Hs00181051_m1	APC	-1,9	up
Hs00183042_m1	MTA1	1,3	down
Hs00153408_m1	MYC	1,9	down
Hs00390028_m1	TCF20	3,0	down
Hs00236077_m1	CEACAM1	3,0	down
Hs00234579_m1	MMP9	3,0	down
Hs00242558_m1	FGFR4	3,1	down
Hs00191018_m1	MTA2	3,7	down

B

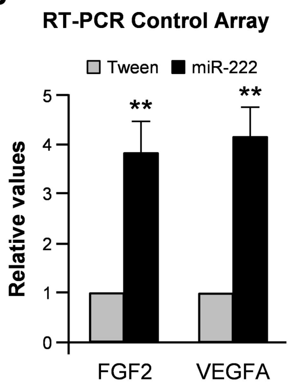

C

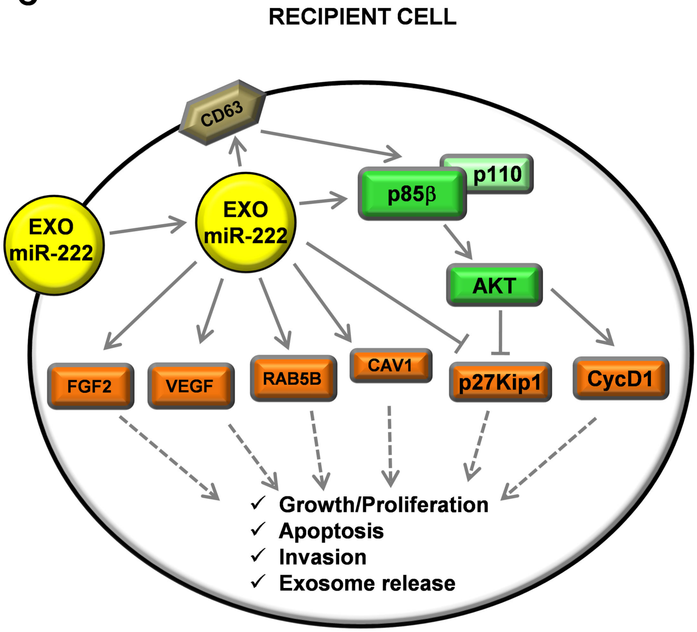

Supplementary Figure 3. . Expression profiling of the exosomal cargo. A) Differentially expressed genes obtained by the TaqMan Array Plate for Human Tumor Metastasis genes in EXO/miR-222 vs EXO/Tween samples. **B)** The expression level of some selected genes modulated in TaqMan Array Plate was confirmed by qRT–PCR. **C)** Schematic illustration of pathways regulated by EXO/miR-222 in melanoma.
